# How to ensure basic competencies in end of life care – a mixed methods study with post-graduate trainees in primary care in Germany

**DOI:** 10.1186/s12904-020-00540-1

**Published:** 2020-03-24

**Authors:** Simon Schwill, Dorothee Reith, Tobias Walter, Peter Engeser, Michel Wensing, Elisabeth Flum, Joachim Szecsenyi, Katja Krug

**Affiliations:** Department of General Practice and Health Services Research, University Hospital Heidelberg, Marsilius Arkaden, INF 130.3, Turm West, 69120 Heidelberg, Germany

**Keywords:** Palliative care, End of life care, Primary care, General practice, Postgraduate education, Residency, Vocational training

## Abstract

**Background:**

Providing end of life care (EoLC) is an important aspect of primary care, which reduces the risk of hospital admission for most patients. However, general practitioners (GPs) seem to have low confidence in their ability to provide EoLC. Little is known about an adequate volume and kind of training in EoLC among GP trainees.

**Methods:**

We performed a before-after comparison in all post-graduate GP trainees who were registered in the vocational training program (KWBW VerbundweiterbildungPLUS). They were offered participation within a two-day seminar focussing on palliative care in 2017. Those who attended the seminar (intervention group I) completed a paper-based questionnaire directly before the intervention (T_1_) and 6 months after (T_2_). None-attendees (group C) were also asked to fill out the questionnaire once. The questionnaire covered previous experiences in palliative care, self-assessment of competencies in EoLC in the organisation of patient care as well as in control of symptoms, attitudes towards death and caring for dying patients and questions about GPs’ role in EoLC.

**Results:**

In total, 294 GP trainees (I: *n* = 219; C: *n* = 75) participated in the study. Of those, more than 90% had previously gained experience in EoLC mainly during vocational training in the hospital rotation. Around a third had previously gained competencies in EoLC in medical school. Between groups I (T_1_) and C no significant differences were observed in socio-demographic characteristics, pre-existing experience or overall expertise. At T_2**,**_ 75% of participants of group I declared they have extended their competencies in EoLC after the intervention and 70% classified the intervention as helpful or very helpful. Overall, they rated their competencies significantly higher than at T_1_ (*p* < 0.01). In detail, competencies in organisation of EoLC and competencies in handling of symptoms significantly improved (p < 0.01). Due to the intervention, 66% could reflect their attitudes towards dying, death and grief and 18% changed their attitudes. Group I highlighted palliative care as one of GPs tasks (Likert 4.47/5, SD 0.75).

**Conclusions:**

The intervention fostered personal competencies, understanding and self-confidence in EoLC among GP trainees. This is crucial for the aim to broadly provide EoLC.

## Background

Providing primary care incorporates end of life care (EoLC): Less than half of the patients in Europe die at home [1] but clearly more would prefer to die at home [2, 3]. Care of family members and primary care physicians (general practitioners (GPs)) determines death at home [4]. A minority of patients (10–15%) need intense EoLC due to complex problems [5, 6]. If these patients wish to die at home, specialised ambulatory palliative care (SAPC) ensures the delivery of EoLC and increases quality of life outside the hospital in Germany [7]. Nevertheless, GPs have shown to reduce hospital admissions by providing generalist palliative care for most of the patients by ensuring coordination with family carers, community health providers and other physicians [8–11]. The involvement and thereby training of GPs in EoLC is crucial, because there are not enough palliative care specialists available [12]. The World Health Organisation encourages a three-step model of EoLC including the concept of an independent specialist training in palliative care [13]. Training of competencies in EoLC differs between countries: Some countries such as Ireland, Poland or the UK have established a separated specialist training in palliative care [14]. Other countries such as Germany offer an additional training in palliative care [15]. Lastly, there are many countries without any additional or specialised training in EoLC [16]. In sum, to ensure comprehensive EoLC is an increasing task for many individuals, and especially for family carers, for community healthcare providers and for GPs.

To provide adequate EoLC in daily GP practice requires various competencies [17, 18]: Firstly, competencies in pain management and dealing with “uncommon” symptoms such as thirst or hunger. Secondly, it requires competencies in the coordination of various healthcare providers. Thirdly, communicational competencies for the appropriate communication with both patients and relatives are needed but comparably challenging because of additional spiritual needs and ethical requests. This fragmentary description underlines the aggravated expectations of patients and relatives faced by all healthcare providers in EoLC, and especially by GPs. In 2017, a qualitative study identified GPs needs and learning preferences in EoLC: It demonstrated that even though clinical practice in EoLC is complex, the exposure to EoLC during training and the training of competencies appear to be inconsistent and inadequate [19]. In accordance to a study from the 1990s, low level of confidence in EoLC among GPs needs to be encouraged [17, 19].

GPs specialist training in Germany is about 5 years and requires hospital and ambulatory rotations. The catalogue of requirements for GPs (*logbuch*) includes knowledge in palliative medicine without further specifications [20]. Since 2003, an additional qualification in palliative medicine (*Zusatzbezeichnung Palliativmedizin*) is eligible for those physicians who successfully specialised in a patient-centred discipline such as anaesthesiology or internal medicine or general practice/family medicine [15]. The course includes a theoretical part (40 h of seminars) and a practical part (12 months of clinical rotation in palliative care which could be replaced by 120 h of case-based seminars instead).

In Germany, vocational training is defined as training on the job and choice of specialisation or sub-specialisation is not regulated [21]. To increase the attractiveness of general practice in face of the looming shortage of GPs in Germany, the first German training program for general practice was founded in 2008 in Baden-Württemberg in the southwest of Germany: The *KWBW VerbundweiterbildungPLUS*© offers a curricular seminar-programme, a structured mentoring-programme and regional clinical rotations all over Baden-Württemberg for postgraduate trainees specialising in general practice (GP trainees) [22]. Participation is voluntarily and financial resources are low which is why each participant can be offered only up to 36 h of seminar-programme per year. The 5-year-curriculum includes EoLC as one main topic.

Until today, studies which evaluate the competencies in EoLC among GP trainees are scarce and could not be found in Germany. According to the scientific programme of the European Association for Palliative Care (EAPC) 2019 world congress, education for postgraduate trainees in general practice is generally rarely addressed [23]. Thus, little is known about an adequate volume and appropriate training in EoLC among GP trainees.

### Aim

Aims of this study were to evaluate self-assessed competencies of GP trainees in treating symptoms as part of EoLC and their attitudes towards palliative care as well as to evaluate the changes after an educational intervention.

## Methods

### Study design

We performed a before-after comparison in post-graduate trainees in general practice. Participants in an educational palliative care intervention (group I) were assessed before (T_1_) and after the intervention (T_2_). Post-graduate trainees not participating in the intervention (non-attendees group C) were assessed once in a cross-sectional survey.

### Setting

All GP trainees registered in the post-graduate training program *KWBW VerbundweiterbildungPLUS* [22, 24] were offered participation within a two-day seminar focussing on palliative care in 2017 (population). In total, there were eight (*n* = 8) equal seminars from January to December 2017 and within this period of intervention GP trainees could choose by themselves whether or not and when to attend. The courses took place in two different seminar-centres in Baden-Wuerttemberg, Germany (Bad Herrenalb, Unterreichenbach). All participants received the full course content. All participants of the training programme work as physicians either part-time or full-time and either in general practice or in hospital rotations (inner medicine, surgery or else). The exposure to EoLC varies from hospital to hospital and from practice to practice.

### Sample

In general, the participation in the study was voluntary. All GP trainees enrolled in the programme were eligible for participation, only those GP trainees involved in the planning and running of this study were excluded. The intervention-group I was recruited from all vocational trainees who voluntarily attended the intervention (a two-day seminar) sometime in 2017. They were addressed personally at the beginning of the seminars for participation in the study. The non-attendees group (C) was formed by all GP trainees who were enrolled in the training programme at the time of the study but did not attend the two-day seminar in 2017. Therefore, non-attendees could be recruited after the intervention-period (2017). They were addressed via email in March 2018.

### Intervention

**Fig. 1:**
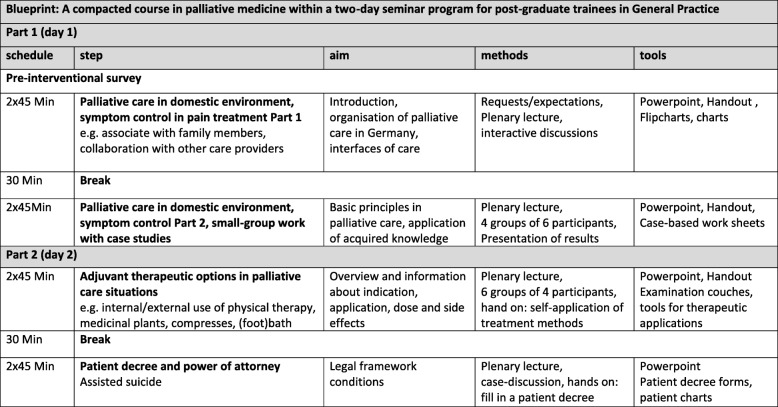
Blueprint: A compacted course in palliative medicine within a two-day seminar program for post-graduate trainees in General Practice

The maximum number of participants on a two-day seminar program was *n* = 30 GP trainees. A tightly scheduled comprehensive course in palliative medicine (intervention) was performed in eight teaching units (each about 45 min) implemented into the two-day seminar program. The main educational objective was to ensure basic knowledge in supporting patients during EoLC in general practices. The hidden curriculum aimed to establish an affirmative attitude towards EoLC. The blueprint of the course contents is shown in Fig. 1: On the first day, palliative care in the domestic environment, organisation of patient care and control of symptoms such as pain or dyspnoea were taught in interactive seminars. The second day focused on adjuvant therapeutic strategies in palliative care and patient’s decree (living and supposed will). An adoption of the course content towards the German National Competency-based Learning Catalogue in Medicine is available in the amendments. In general, seminars included case-based discussions with example cases of the lecturers as well as personal cases of the trainees. In total, three lecturers participated, each with more than 20 years of professional experience as a GP, additional qualification in palliative care, still working in primary care and member of a SAPC team. Additionally, one lecturer holds a degree as Master of Medical Education as well as training in naturopathic treatments; the other two lecturers initiated “PAMINO” - an initiative for palliative care in southern Germany [25]. In total, the intervention took place eight times without any organisational difficulties in 2017. After the first seminar in January 2017, minor modifications were required; the intervention could be performed 7 times as shown in Fig. 1. Part one and part two on the first day were always given by the same lecturer. Part three and four were performed by either one of two lecturers.

### Data collection

Group I was requested to complete a paper-based questionnaire directly before the intervention (T_1_). Six to 8 months after the intervention (T_2_) they received an evaluation questionnaire via mail including a pre-paid return envelope (follow up). A reminder was sent via email 2 months later. Group C received an invitation to participate in an online survey (SurveyMonkey, SurveyMonkey Europe UC, Dublin, Ireland) in March 2018 for a period of 8 weeks. Reminder emails were sent three and 5 weeks after the initial invitation. There was no follow up within group C. Data collection was completed in July 2018. This study was approved by the ethics committee of the University of Heidelberg (approval number S 570/2015) in advance. All participants were informed about the study and provided signed informed consent to participate.

### Outcomes

Competencies in EoLC were assessed in 20 items about symptom control and organisation of patientcare, which participants rated on a five-point-Likert-scale. Attitudes towards EoLC were assessed by dichotomous (yes/no) question whether or not they have changed their attitude and an open-text section to reveal type of change and personal reflexion as well as increase of knowledge due to the intervention.

### Measures

The questionnaire was developed by the authors on the basis of comprehensive literature analysis as well as hands-on experience in palliative care. All three variations of the questionnaire were revised by think-aloud technique and piloted, which led to some modifications and additional explanations. The main part included 20 items regarding personal skills in which participants were asked to rate their competencies in EoLC, especially in the organisation of patient care as well as in symptom control on a 5-point Likert scale (1 = none to 5 = maximum). In adoption to the different points of time and different groups further questions were necessary in the survey at T_2_ and for group C.

The survey at T_1_ used a paper-based questionnaire containing 20 items of the main part and 5 items regarding previous experience and additional qualification in palliative care. The follow-up survey at T_2_ used a questionnaire containing 34 items. The questions were designed corresponding to the first questionnaire at T_1_ (main body). In addition, we assessed if participants acquired expertise by attending the two-day seminar program as well as how helpful the two-day seminar program was evaluated regarding practical work including free text sections. Furthermore, participants had the opportunity to name the three most important aspects of the two-day seminar program in a free-text section. Two dichotomous questions surveyed possible reflections or changes in attitude regarding death and caring for dying patients, with a subsequent free-text for further explanation. The questionnaire for group C contained 33 items: It included the 25 items of the T_1_ survey as well as five questions regarding sociodemographic data (gender, date of birth, year of residency, rotation, date of joining *KWBW VerbundweiterbildungPLUS*). At follow-up (T_2_) and for group C, 3 items were added asking participants to rate palliative care as a responsibility of GPs.

### Data analysis

For analysis, all data were pseudonymously entered into SPSS (IBM Statistics, Version 25). Characteristics of trainees were summarised in terms of frequencies of categories, means with standard deviation and medians with interquartile range for continuous variables. Differences in frequencies between the groups were analysed using chi-square tests; differences between T_1_ and T_2_ were analysed using t-tests for dependent samples and McNemar-tests. Free-text sections were screened with qualitative content analysis by two independent researchers experienced with qualitative analyses. At first, all free-text sections were independently summarised into codes. Secondly, categories were developed inductively from the codes. Finally, researchers consented on categories and coding with an uninvolved third researcher. No specific software-tool was used. Translation of qualitative data was validated by four independent researchers.

## Results

In total, 294 GP trainees (I: *n* = 219; C: *n* = 75) participated in the study. *N* = 224 (median per course 29, range 23–32) trainees participated in 8 independent courses. Two participants were involved in the conception of the study and therefore had to be excluded from the survey. 219 pre-intervention questionnaires (response rate 98.6%, n = 219/222) were completed and collected. 153 post-intervention questionnaires (response rate 68.9%, *n* = 153/219) were returned. One questionnaire returned after closure of survey period and had to be excluded from analysis. 183 general practice trainees were invited for group C, 75 participated (response rate 40.9%).

### Socio-demographic characteristics of participants

Sociodemographic data of group I is shown in Table 1: The participants in group I were mostly female (*n* = 143/219, 65.3%). In group C, 76% (*n* = 57/75) were female. Mean age was 36.6 years (SD 6.8) in group I in comparison with 36.7 years (SD 6.9) in group C (*p* = 0.74.). Statistical analysis showed no significant differences between responders and non-responders at T_2_ in group I besides distribution between the sexes: Women completed the post-intervention survey more frequently than men (*p* < 0.05).

**Table 1:** Socio-demographic characteristics and experiences in end-of-life care of post-graduate trainees in general practice

	Non-attendees group (C)	Intervention group (I)		p (I(T_1_):C)
	*n* = 75	T_1_ n = 219	T_2_ n = 153	
Gender				
female, n (%)	57 (76.0)	143 (65.3)	106 (69.3)	.09^a^
male, n (%)	12 (16.0)	55 (25.1)	32 (20.9)	
n.a., n (%)	6 (8.0)	21 (9.6)	15 (9.8)	
Age in years M (SD) [range]	36.7 (6.9) [26–61]	36.3 (6.8) [26–57]		n.s.^b^
Preexisting experiences in palliative care, % (n)	82.7 (62)	86.3 (189)	74.5 (114)	n.s.^a^
Experience gained, n (%)				
- In hospital	57 (91.9)	178 (94.7)	29 (25.4)	n.s.^a^
- In general practice	27 (43)	60 (31.9)	89 (78.1)	n.s.^a^
- Personally (e.g. family)	3 (21)	35 (18.6)	6 (5.3)	n.s.^a^
- other	3 (4.8)	13 (6.9)	8 (7.0)	n.s.^a^

*M* mean, *SD* standard deviation, ^a^χ2 test, ^b^ t-test

### Pre-existing experiences in end of life care

86.3% (189/219) of the participants had already experienced end of life care, mainly during post-graduate education while working in hospitals (94.7%). The response “other experiences” was most frequently specified as “palliative care unit”, followed by “hospice/nursing home” and SAPC. Three participants had already passed the additional qualification in palliative care, 61 intended to pass the qualification at the time of the survey, of which 12 had already started with the necessary courses.

Between groups I (T_1_) and C there were no significant differences in socio-demographic characteristics, pre-existing experiences, existence of or motivation to achieve the additional qualification of palliative care or overall expertise. In group C, other experiences were mainly made in hospices or in the SAPC. Expertise was significantly more often acquired in personal surroundings (e.g. family) as well as “other (e.g. hospice)” when compared to T_1_ in group I (*p* < 0.05). Participants in group C self-rated their organisational expertise significantly lower in the items “collaboration with other care providers” (*p* < 0.05) and “involvement of family members” (*p* < 0.05). A comparison of T1 and C is shown in Table 2.

**Table 2:** Comparison between T_1_ (pre-interventional survey) and non-attendees group (uncorrected single tests)

	Intervention group (I) T_1_n=219	Non-attendees group (C) n=75	p (I(T_1_):C)
**Acquired experiences in palliative care** n (%)	189 (86.3)	62 (82.7)	.44
- in a hospital	178 (94.7)	57 (91.9)	.43
- in a GP practice	60 (31.9)	27 (43.5)	.10
- in personal environment	35 (18.6)	13 (21.0)	.68
- other	13 (6.9)	3 (4.8)	.77
**Strive for the additional specialisation in palliative care** n (%)	61 (29.0)	18 (24.0)	.40
- already started with courses n (%)	12 (19.0)	7 (38.9)	.08
- already received additional specialization n (%)	3 (5.7)	2 (11.1)	.60
**Acquired expertise in palliative care** M (SD)	2.9 (0.8)	2.8 (1.0)	.73
- in Medical school n (%)	68 (32.1)	14 (20.3)	.06
- Self-study n (%)	36 (17.0)	15 (21.7)	.37
- in a hospital n (%)	185 (87.3)	61 (88.4)	.80
- in a GP practice n (%)	59 (27.8)	26 (37.7)	.12
- in personal environment n (%)	35 (16.5)	19 (27.5)	<.05
- professional training n (%)	32 (15.1)	12 (17.4)	.65
- other n (%)	11 (5.2)	9 (13.0)	<.05
**Expertise in organization of palliative care situations regarding** M (SD)			
- medical treatment in domestic environment	2.5 (1.0)	2.3 (1.0)	.11
- collaboration with other care providers	2.5 (1.1)	2.2 (1.0)	<.05
- involvement of family members	3.2 (1.0)	2.8 (1.1)	<.05
- Integration of other ambulant health care providers	2.7 (1.1)	2.6 (1.3)	.52
- Hospitalization of patients (in a palliative care unit or hospice)	2.8 (1.1)	2.7 (1.1)	.24
**Expertise in handling of following items in palliative care situations** M (SD)			
- Pain	3.3 (0.8)	3.2 (1.0)	.23
- Gastrointestinal symptoms	3.1 (0.8)	3.0 (0.9)	.24
- Dyspnoea	3.3 (0.9)	3.1 (1.0)	.11
- Anxiety and agitation	3.2 (0.9)	3.0 (1.0)	.08
- Delirium	2.8 (1.0)	2.5 (1.0)	<.05
- Fatigue	2.2 (0.8)	2.1 (1.0)	.26
- Fluid intake	3.2 (0.9)	3.1 (1.1)	.61
- Nutrition	3.0 (0.9)	2.9 (1.1)	.57
- Psychological problems	2.9 (0.9)	2.6 (1.0)	<.01
- Spiritual issues	2.7 (1.1)	2.7 (1.2)	.69
- Ethical issues	3.1 (1.0)	2.7 (1.1)	<.05
- Family members and social environment	3.4 (0.9)	3.1 (1.0)	<.05
- Grief	3.3 (0.9)	2.9 (0.9)	<.01

^**a**^dependent t-test, *M* mean, *SD* standard deviation, 1 Likert-Scale (min. 1 to 5 max.), 2 expertise acquired since the intervention (within 6–8 months)

### Pre-existing competencies in end of life care

Competence in control of symptoms was perceived as significantly lower by participants of group C in five of overall 13 assessed symptoms compared to group I at baseline: control of delirium, psychological problems, spiritual and ethnic questions, managing family members and social environment as well as dealing with people’s grief (Table 3).

**Table 3:** Expertise in end-of-life care of post-graduate trainees in general practice

	Intervention group (I)		*p*^a^
	T_1_*n* = 219	T_2_*n* = 153	
**Overall level of competency**^**b**^**M (SD)**	2.9 (±0.8)	3.1 (±0.7)	<.01
**Competencies acquired in, % (n)**			
- hospital	84.5 (185)	15.7^c^ (24)	
- general practice	26.9 (59)	40.5^c^ (62)	
- personal environment	16 (35)	3.3^c^ (5)	
- postgraduate training	14.6 (32)	6.5^c^ (10)	
- medical school	31.1 (68)	5.2^c^ (8)	
- self-study	16.4 (36)	15.7^c^ (24)	
- other	11 (5)		
- intervention (two day seminar program)		22.9^c^ (35)	
**Organisation of**^**b**^**M (SD)**			
- treatment in domestic environment	2.5 (±1.0)	3.0 (±0.9)	<.01
- collaboration with other care providers	2.5 (±1.0)	3.0 (±0.9)	<.01
- involvement of family members	3.2 (±1.0)	3.5 (±0.9)	<.01
- integration of other ambulant health care providers	2.7 (± 1.1)	3.2 (±1.1)	<.01
- hospitalisation of patients (to a palliative care unit or hospice)	2.8 (±1,1)	3.1 (±1.1)	<.01
**Handling of**^**b**^**M (SD)**			
- pain	3.3 (±0.8)	3.6 (±0.8)	<.01
- gastrointestinal symptoms	3.1 (±0.8)	3.5 (±0.8)	<.01
- dyspnoea	3.3 (±0.9)	3.5(±0.8)	<.01
- anxiety and agitation	3.2 (±0.9)	3.3 (±0.8)	<.05
- delirium	2.8 (±1.0)	2.8 (±0.8)	.05
- fatigue	2.2 (±0.8)	2.4 (±0.8)	<.01
- fluid intake	3.2 (±0.9)	3.5 (±0.7)	<.01
- nutrition	3.0 (±1.0)	3.3 (±0.8)	<.01
- psychological problems	2.9 (±0.9)	3.1 (±0.9)	<.05
- spiritual questions	2.7 (±1.1)	3.1 (±1.0)	<.01
- ethical questions	3.1 (±1.0)	3.3 (±0.9)	<.01
- family members and social environment	3.4 (±0.9)	3.6 (±0.7)	<.01
- people in grief	3.3 (±0.9)	3.5 (±0.8)	<.01

^**a**^ dependent t-test, *M* mean, *SD* standard deviation, ^**b**^ Likert-Scale (min. 1 to 5 max.), ^c^ expertise acquired since the intervention (within 6–8 months)

### Changes in the intervention group

74.5% (*n* = 114/153) of participants gained further experiences in palliative care after attending the compacted intervention. In contrast to T_1_, most experiences were gained in primary care practices (78.1%, *n* = 89/114). As in the pre-interventional survey, “other experiences” were made in nursing homes, hospices, SAPC and palliative care units. 22.9% (*n* = 35/153) mentioned to have gained competencies in end-of-life care by the intervention. 107 out of 153 post-graduate trainees (69.9%) evaluated the participation of the two-day seminar program as helpful or very helpful regarding their practical work. In T_2_, participants ranked their overall expertise in end-of-life care significantly higher than before (*p* < 0.01). Apart from “dealing with delirium”, all competencies in the survey were rated significantly higher after the intervention. Detailed results are shown in Table 3.

#### Most important content of the course

In group I, 69.9% (*n* = 107/153) filled in the free-text field about the “three aspects regarding palliative care during the two-day seminar most important to you”. Data was analysed and codes were assigned to 7 categories: Medical control of symptoms, adjuvant therapies, communication, legal framework conditions, organisation of care, change of focus and others (Table 4).

**Table 4:** Qualitative analysis of the most important contents of the intervention (7 categories, 38 codes)

The most important 3 aspects of the course (*n* = 107/153 general practice trainees):	
**category**	**code**
**Control of symptoms (with medication)**	Treatment of pain / opiate therapy
	Treatment of neurological symptoms (fear, delirium)
	Dealing with dyspnoea
	Dealing with nausea
	Dealing with chronic wounds
	Dealing with sense of hunger / nutrition
	Dealing with sense of thirst / fluid therapy
**adjuvant therapies**	Oral hygiene
	Aroma therapy
	Thermotherapy
**communication**	Involvement of relatives
	To ensure openness with the patient
	To permit ethical discussions
	To respect patients’ fears
	To address spiritual needs
	Collaboration of GP and hospital
**legal framework conditions**	Patient’s decree (living will)
	Attorney for personal care
	Supposed will
**organisation of care**	Enable death at home by ambulatory end of life care
	Use of hospices
	To ensure personal setting / framework at home
	To write a treatment plan
	If necessary, integration of specialized ambulatory palliative care
**change of focus**	Personal approach: Patient’s (living/supposed) will and needs are pivotal
	Focus on psychosocial support of the patient
	There is no “golden path”
	To question treatment and intentions.
	There is a lot to do at the end of life.
**others**	The use of practical case studies
	Reduced fear with end of life care issues
	To experience that palliative medicine is an interesting working field of medicine
	The personal experience of adjuvant therapies
	To realise that level of knowledge needs to be extended.
	The practical long-time experience of the lecturers
	The lecturers’ attitude served as a role model
	To learn that self-care for the treating physician is no egoism
	The reflection of the personal medical action

Most frequently, medical control of symptoms was mentioned, particularly with regard to pain treatment and handling of opioids as well as neurological symptoms (anxiety, delirium or similar), dyspnoea, nausea, chronic wounds, nutrition and handling of appetite/hunger as well as fluid therapy and handling of thirst. One participant commented on an aspect most important to him:

*“What I am allowed to do as a GP to ease pain, dyspnoea, agitation and anxiety of a palliative patient.” (#65)*

Frequently mentioned were learning about and the use of adjuvant therapies and particularly referring to oral care, aroma therapy and thermotherapy. Thereby having the opportunity of personal experience was highly appreciated:

*“The many aspects of oral care” (#7),*

*“tips and tricks like the frozen favourite drink” (#40),*

*“to use alternative and naturopathic ways of symptom relief” (#32),*

*“supporting features like aroma therapy are not taught in hospitals and not often used” (#64).*

Participants mentioned acquiring communication skills as useful during the course: In particular they had learned to involve relatives, to foster a culture of openness with patients and to allow and address ethical questions and spirituality, to take away fears and to improve the interaction between hospitals and GPs.

“*Focusing on collaboration with family members” (#14),*

*“Handling of ethical and spiritual questions” (#72),*

*“Not being afraid of an open communication with patients” (#87).*

Other participants emphasised having gained knowledge about patient’s decree (living will), power of attorney for personal care and handling of patient’s supposed will, summarized in legal framework conditions:

*“Clarification of the terms patient decree, living will and attorney for personal care, legal conditions and patient autonomy” (#69),*

*“The legal situation of taking (therapeutic) measures or refraining measures” (#100).*

To some participants organisation of (end-of-life) care was considered an important content of the course such as the modalities of admission to a hospice or writing a care/provision plan. Furthermore, the fundamental role of ambulatory end-of-life care to organise the personal setting and thereby enable dying at home was mentioned:

*“Possibilities of ambulatory palliative care for patients” (#10),*

*“What palliative medicine can and cannot provide in a GP setting” (#44),*

*“Making a plan as a GP” (#93).*

Participants described a change of focus due to the clarification of a personalised approach during the course. They mentioned the patient’s will to be pivotal and the idea of an individual approach tailored to the needs of each patient as well as the psychosocial support (accompany patients):

*“Accompany patients instead of prolonging their life at any cost” (#28),*

*“Meaning well is not automatically doing well: there is no golden path. The patient and his or her needs and requirements determine what and how things need to be done” (#9),*

*“When life ends, a lot can be done for patients” (#13).*

Finally, GPs in training extolled hands-on training and the opportunities for personal experience. Particular emphasis was put on the professional demeanour and practical experience of the lecturers. Participants stated to have lost their fear of contact and said to have discovered palliative medicine as an interesting field of expertise:

*“(Palliative Medicine,) An interesting field of occupation in the professional life of a GP” (#13),*

*“Strengthening self-confidence with regard to dealing with dying patients and the attitude of the lecturers” (#50).*

In total, *n* = 3 participants described the course as basic rather than helpful for themselves but as a refresher. They declared having extensive previous experience in palliative care (2 years in geriatrics with palliative ward, certificate additional qualification on palliative care (*n* = 2)).

#### Attitudes towards end of life care

Group I was asked if they had reflected their own attitude towards dying, death and grief because of the course and if they had changed their attitude. In total, 66% (*n* = 101/153) could reflect on their attitude because of the course and *n* = 28 (18.3%) had changed their attitude because of the intervention. The majority of those specified their change of attitude in the free-text section of the questionnaire. The findings could be summarised as increased understanding for dying patients and their relatives, enhancement of self-confidence to deal with palliative patients and an overall growth of awareness and interest for EoLC (Table 5).

**Table 5:** Quotes of general practice trainees: Change of attitude towards dying, death and grief because of the intervention

general practice trainees:	
“I became aware of how important end of life care is for both, the patient as well as their relatives.”	
“I want to improve the quality of end of life care.”	
“I want to support patients to leave in dignity and without sorrows.”	
“I want to identify and value the will and needs of my patients.”	
“I consider the combination of a multimodal therapy within a multi-disciplinary team as best option” (for end of life care).	
“I became aware of an increased need for physical contact in dying patients.”	
“I do not want to judge relatives’ grief as being pathological too early”	
“Alternative therapies in oral hygiene and aroma therapy are helpful.”	
“I feel more self-confident.”	
“Sometimes easy things such as oral hygiene make the slight difference”	
“I became aware of end of life care to be a task in general practice.”	
“I became interested in palliative medicine.”	
“Now, I do have more understanding and can be more empathic towards patients and their relatives.”	
“If they want, general practitioners can support patients in their wish to die at home”	
“Palliative medicine is comprehensive.”	
“Now I can imagine letting patients go (= let them die).”	
“I became aware of a special patient-doctor relationship at the end of life.”	
“I want to accept the patient’s will and autonomy in any situation, even if it is not reasonable from a medical point of view.”	
“I want to analyse the problems of dying patients in detail and want to question decisions in therapy more often.”	
“I obtained a better understanding of the various problems”	
“I reflected thoroughly relative’s options (in end of life care). How would I like to die?”	

#### Additional qualification

24% (*n* = 18/75) of group C compared to 28.5% (*n* = 61/214) of group I at T_1_ intended further specialisation in palliative care (p = n.s.). Of these, 38.9% (*n* = 7/18) of group C and 19% (*n* = 12/61) of group I had already started the course. After the compacted intervention, three participants had started with the courses. Analysis showed that one trainee had achieved the qualification since the intervention. Comparing T_1_ (28.5%, *n* = 61/219) and T_2_ (19.9%, *n* = 30/153), significantly less participants aimed to achieve the additional qualification (*p* < 0.05).

### End-of-life care – a task for general practitioners?

EoLC was rather perceived as part of general practice in group I than in group C. Group I rated the statement “Palliative care is one of my tasks as a GP” with a mean of 4.47 (SD 0.75) on a 5-point Likert scale in contrast to group C who rated it with 4.11 (SD 0.83). “Palliative care is a fundamental part of general practice” was rated with a mean 4.37 (SD 0.84) in group I but with 3.92 (SD 1.0) in group C. Finally, “palliative care should primarily be performed by GPs” was rated with a mean 3.56 (SD 0.98) in group I and with 2.88 (SD 1.1) in group C.

## Discussion

In our study, *n* = 294 GP trainees self-rated their competencies in EoLC to be just average and that their organisational expertise in EoLC is inferior to their medical competencies. However, 6 months after a compacted intervention the vast majority of participants declared they have extended their competencies in EoLC and defined EoLC as one of their tasks as a GP and a fundamental part of general practice. Finally, competencies in both organisation of EoLC and handling of symptoms significantly improved on individual level and in two third of the participants the intervention induced a reflection of personal attitudes towards dying, death and grief.

To the best of our knowledge this is the first study to assess subjective competencies in EoLC among GP trainees in Germany. The results suggest insufficient teaching in EoLC at medical schools and outline the hospital-rotation in internal medicine as first learning situation in EoLC for post-graduate trainees. Since 2014, graduates from German medical schools benefit from palliative care as a cross-sectional subject with obligatory examination which was found in 2009 [26]. However, the vast majority of participants in the study graduated earlier than 2014. Another study from 2015 explored key areas of EoLC and demanded an early acquisition of skills in EoLC within the medial career [27]. To us, these findings underline that GPs’ presence in teaching palliative care in medical schools should be enlarged and the collaboration between hospitals and GPs could be improved for both training of medical students and GP trainees.

The intervention successfully fostered a learning process in which post-graduate trainees increased their competencies in EoLC. Participants felt more confident in organisation of EoLC as well as in handling of symptoms. This is remarkable because the intervention itself was comparably short (6 h). It therefore initiated a learning process which went on within the clinical / practical rotation. Possibly, the main effect of the short-time intervention is an increase in self-confidence due to a reactivation of competencies and the induction of an awareness of EoLC in daily practice. A study from Canada from 2000 described high self-confidence in EoLC among GP trainees [28]. Our results show that after the intervention, 40.5% (*n* = 62/153) of participants gained experience in the practice. Some might interpret that the participants had not previously reflected and thereby had not previously accepted their task to get involved into ambulatory EoLC. A study in postgraduate trainees on intensive care units could show that self-confidence is a very important factor for the delivery of appropriate EoLC [29]. In accordance with studies showing a lack of confidence among GPs in EoLC [19, 30–33], the intervention might have triggered GP trainees self-confidence.

The intervention was helpful for the targeted learning-group: It could increase an understanding for EoLC and induced the GP trainee’s reflection of EoLC in 66% (*n* = 101/153). One out of five participants changed his/her attitude towards EoLC in a preferable manner such as an increased understanding for dying patients and their relatives and an overall growth of awareness and interest for end-of-life care. Therefore, one main effect of such intervention could be the change of attitude. We realised that the intervention had clarified GP trainees’ assumptions about EoLC: Significantly less participants than expected aimed to achieve the additional qualification after attending the intervention (*p* < 0.05). This supports the effect of the intervention to give an overview about EoLC, which was achieved and thereby the need for further training in EoLC was satisfied in these participants. On the other hand, the participants may have experienced a realistic point of view towards the efforts of the additional qualification in palliative medicine and the demands of EoLC, which have already been described 30 years ago [34]. Perhaps training in EoLC (for GPs) is as much about attitudes as well as about competencies.

In summary, there is no doubt that competencies in EoLC are mandatory for GPs [35]. EoLC is a topic under direct supervision of the World Health Organisation and affects people from all societies within the world: Provision of EoLC is equalised to quality of life and the term quality of death arises [36]. EoLC is part of the political debate as well as of ethical discussions and it is just about the (basic) content and quality we need to agree upon [13]. Internationally, there is a lack of literature on the education of competencies in EoLC in both post-graduate trainees and especially in GP trainees: There are few published curricula such as from 2016 [37] and the questions of how and how much of teaching of EoLC is necessary during specialisation in general practice. If for example communicational skills in EoLC should be trained or if an intense course on EoLC is developed a well-planned curriculum based on previous experiences is mandatory [38, 39]. A recent study, which was not available in 2016 when we had planned our study, presented a list of necessary learning objectives and goals for EoLC [40]. The compacted intervention in our study was shown to fulfil the targeted learning objectives - to ensure basic knowledge in EoLC and to establish an affirmative attitude towards EoLC – and was rated as helpful which is why it might be applicable and sufficient as basic as well as refresher training for experienced as well as upcoming GPs in other places, too. Altogether, one aim of the curriculum of the GP specialist training-programme KWBW VerbundweiterbildungPLUS could be achieved. To empower GP trainees in competencies in EoLC is pivotal for the comprehensive provision of EoLC.

### Limitations

In general, due to the voluntary participation in the study there are non-follow-up’s whereas the response rate after the intervention was high (response rate 68.9%, *n* = 153/219). Participation in the training-programme in general as well as in the intervention were voluntary, therefore a selection bias could not be excluded. Due to voluntary participation a randomisation was not applicable, but there were no statistically relevant differences between the intervention-group and the control, even in regard to the long time period of the study. The intervention fostered the gain of competencies but the educational intervention was not the only effect on the trainees within the period of 6 months after the intervention. Thereby it is not possible to quantify the effect of the intervention itself. A direct survey after the intervention was not applicable because of the voluntary participation in the study and the aim to pronounce long time learning effects. The questionnaires were solely developed for the current study and validated and piloted by think aloud technique whereas further validation was not possible. In this study, participants self-rated their skills on a 5-point Likert scale. A testing of competencies (written and/or oral and/or practical such as directly observed procedures) could not be implemented. There is no written examination for post-graduate trainees in Germany and intention for voluntary participation was expected to be low.

## Conclusions

The intervention had successfully fostered a gain of personal competencies in EoLC within low-resource circumstances. Thereby, basic competencies in EoLC in participating trainees and even more important, an understanding and interest in EoLC among post-graduate trainees in general practice could be achieved. This is pivotal for the aim to broadly provide EoLC. Future hands-on experience and studies might show how much the upcoming generation of GPs will become actively involved in EoLC and will help the patients to follow their wish to peacefully die at home.

## Supplementary information

**Additional file 1.**

## Data Availability

The datasets used and/or analysed during the current study are available from the corresponding author on reasonable request.

## Abbreviations

GP: General practice
GPs: General practitioners
KWBW: *Kompetenzzentrum Weiterbildung Baden-Württemberg (GERMAN) =* Competence Center Postgraduate Education Baden-Württemberg
SAPC: Specialised ambulatory palliative care
SAPV: *Spezialisierte Ambulante Palliativ-Versorgung (GERMAN) =* Specialised ambulatory palliative care
