# Supplementary material for: How to ensure basic competencies in end of life care – a mixed methods study with post-graduate trainees in primary care in Germany
Source: BMC Palliat Care. 2020 Mar 24;19:36. doi: 10.1186/s12904-020-00540-1 (PMC7093985; doi:10.1186/s12904-020-00540-1)
Supplement: Supplementary file 1 — Additional file 1. [file 12904_2020_540_MOESM1_ESM.docx]

## Amendments

### Table 6

**Aspired learning objectives of the German National Competency-based Learning Objectives Catalogue in Medicine:**

Day 1:

- To consider interactions between decisions and social environment of patients e.g. in palliative care (14c.2.6.5)
- to communicate truly and emphatic with dying patients and their family members (14c.3.2.11)
- to explain and criticize principles of palliative care und palliative treatment e.g. symptom control and acceptance of death as a part of life, end-of-life decision (16.1.1.3)
- to guarantee that patients get a sufficient terminal care and that family members get all the appropriate amount of support (5.4.1.4)
- to involve all relevant characters and specialties in decision making (8.1.1.1/11.4.2.3)

Day 2:

- to discuss concepts and methods of classic naturopathic treatment regarding efficacy and risks (16.9.1.12)
- to apply ethical and legal principles in changing therapeutic objectives and limitations including artificial nutrition (18.3.4.5)
- to apply ethical and legal principles in symptom control that could potentially cause a shortening of life (18.3.4.6)
- to talk about the subject of living will with patients (14c.2.8.11)
- to know and consider legal conditions and obligations as well as ethical principles of medical action (11.1.2)
- to be familiar with ethical challenges at the end of life (18.3.4)
